# Developing Health Management Competency for Digital Health Transformation: Protocol for a Qualitative Study

**DOI:** 10.2196/51884

**Published:** 2023-11-03

**Authors:** Mark Brommeyer, Zhanming Liang, Maxine Whittaker, Mark Mackay

**Affiliations:** 1 College of Public Health, Medical and Veterinary Science James Cook University Townsville Australia

**Keywords:** health care management, health service manager, digital health, health informatics, competency, workforce development, innovation, research protocol, informatics, manager, managers, service, services, delivery, organization, organizational, workforce, management, managerial, qualification, qualifications, focus group, focus groups, interview, interviews, scoping, review methods, review methodology

## Abstract

**Background:**

Globally, the health care system is experiencing a period of rapid and radical change. In response, innovative service models have been adopted for the delivery of high-quality care that require a health workforce with skills to support transformation and new ways of working.

**Objective:**

The aim of this research protocol is to describe research that will contribute to developing the capability of health service managers in the digital health era and enabling digital transformation within the Australian health care environment. It also explains the process of preparing and finalizing the research design and methodologies by seeking answers to the following three research questions: (1) To what extent can the existing health service management and digital health competency frameworks guide the development of competence for health service managers in understanding and managing in the digital health space? (2) What are the competencies that are necessary for health service managers to acquire in order to effectively work with and manage in the digital health context? (3) What are the key factors that enable and inhibit health service managers to develop and demonstrate digital health competence in the workplace?

**Methods:**

The study has adopted a qualitative approach, guided by the empirically validated management competency identification process, using four steps: (1) health management and digital health competency mapping, (2) scoping review of literature and policy analysis, (3) focus group discussions with health service managers, and (4) semistructured interviews with digital health leaders. The first 2 steps were to confirm the need for updating the current health service management curriculum to address changing competency requirements of health service managers in the digital health context.

**Results:**

Two initial steps have been completed confirming the significance of the study and study design. Step 1, competency mapping, found that nearly half of the digital competencies were only partially or not addressed at all by the health management competency framework. The scoping review articulated the competencies health service managers need to effectively demonstrate digital health competence in the workplace. The findings effectively support the importance of the current research and also the appropriateness of the proposed steps 3 and 4 in answering the research questions and achieving the research aim.

**Conclusions:**

This study will provide insights into the health service management workforce performance and development needs for digital health and inform credentialing and professional development requirements. This will guide health service managers in leading and managing the adoption and implementation of digital health as a contemporary tool for health care delivery. The study will develop an in-depth understanding of Australian health service managers’ experiences and views. This research process could be applied in other contexts, noting that the results need contextualization to individual country jurisdictions and environments.

**International Registered Report Identifier (IRRID):**

DERR1-10.2196/51884

## Introduction

### Definitions

For the purpose of this protocol paper, the following definitions are being applied: (1) *health services managers* are the “professional managers charged with the responsibility of leading and managing individual health care services, large healthcare organisations and the overall health system” [1]; (2) *digital health* is “an umbrella term referring to a range of technologies that can be used to treat patients and collect and share a person’s health information” [2]; and (3) *digital health services* can be seen as the use of digital tools and processes to provide and support the delivery of health care in a digital society [3].

### Background

#### Overview

The health care system is experiencing a period of rapid and radical change. From a global perspective, the aging population, an escalating burden of chronic disease and pandemics, and increasing community expectations are all placing growing demands on health care services [4]. As a response, innovative service models have been adopted for the delivery of high-quality care to meet this burgeoning need and to improve the efficiency, value, and safety of health service provision [5]. Innovation has included the adoption and implementation of digital health solutions, which were not possible a generation ago. Hence, a health workforce with the requisite skills to support such transformation requires new thinking and new ways of working.

#### Digital Health Transformation Has Been Witnessed

Digital health services have been shown as crucial to developing just-in-time, tailored, and targeted health care that is accessible, affordable, appropriate, and sustainable [6]. Digital health has become “an umbrella term referring to a range of technologies that can be used to treat patients and collect and share a person’s health information” [2]. To ensure success in the digital transformation of health services, digital health solutions need to be designed, developed, and delivered in an effective, efficient, ethical, and evidence-based manner under good governance and management [7-10].

The COVID-19 pandemic saw an acceleration in the uptake of digital health solutions globally. This required health care practitioners and providers to adapt to new ways of working, with the ever-present social distancing and travel restriction requirements becoming commonplace. As an example of the accelerated uptake during the pandemic, digital-first strategies, remote monitoring, and digital care approaches were quickly implemented to enable continuity of health care service without physical interactions [11]. Across Europe, digital health systems enabled critical support of public health policies [12,13] improving the monitoring, surveillance, information, and communication regarding COVID-19 requirements and its impact on health care, as well as the recording and monitoring of vaccination bookings [5]. In the United States, “technology innovations and policy prescriptions” [14] and “right-sizing of regulation” [14] were implemented to support the rapid adoption of innovative, digital health technologies. Furthermore, the web-based medical visit requirements under the HIPAA (Health Insurance Portability and Accountability Act) were also recalibrated [14]. Additionally, the Australian federal government’s responses to the pandemic included implementing the necessary policy and funding requirements for innovative digital health solutions to be deployed across the country [15].

#### Demands on a Skilled Workforce

In response to the fast adoption of digital health technologies, a competent health care workforce with capabilities and confidence in implementing and integrating digital health technologies into health service delivery is essential [6,16]. This includes the management and leadership of health care organizations in the digital health context. Some efforts in addressing the required workforce competencies have occurred. For example, the United Kingdom’s National Health Service [17] has responded by instantiating the Graduate Management Training Scheme for leadership development that includes a Health Informatics specialization, which leads to a postgraduate diploma in data analytics.

In Australia, the Australian Digital Health Agency [18] has developed the National Digital Health Workforce and Education Roadmap and recently noted that “there has been significant interest in emerging literature that examines the future of work and the impact digital technologies will have on the health workforce, or indeed are having. However, there has not been as much concentration on the planning, programs and strategies required to improve the digital health literacy of the health workforce.” Therefore, in the Australian context, it is imperative to focus on the digital health workforce, management competency, and capability development, with contemporary and contextual training that is relevant and responsive to the current health care environment.

#### Competency, Context, and Performance

How health service managers plan and manage the increasingly complex digital tools in an ever-changing environment, along with the resultant rise in digital health literacy, requires capabilities and contextual competencies to ensure these new technologies are best used to inform both strategic and operational decision-making [19-23]. This is supported by the growing evidence highlighting the importance of the investment required to develop a competent health service management workforce, including through academic credentialing and professional association certifications [24].

Although various management competency frameworks have been established to guide the development of management competence for health service managers [25,26], whether these frameworks have adequately captured the competency requirements for health service managers in managing the necessary, fast-paced change and digital transformation of health care is not evident. This highlights a need to articulate the competencies that are necessary for health service managers to acquire to effectively work with and manage in the digital health context. Therefore, confirming the changing competency requirements for health service managers in managing and leading digital health transformation in health care is warranted. This research is being undertaken in the Australian health care setting to contextualize and inform national frameworks, system enablers, and factors that support Australian health service managers in the digital context.

A review of the literature confirmed that the development of competence is context-sensitive [27,28], with performance improvement resulting from competency development. To improve a health service manager’s capability in facilitating and implementing digital health transformation, it is desirable that health service managers develop relevant competencies. Providing an environment that enables managers to fulfill their responsibilities is equally important [29].

### Preparatory Phase (Phase 1)

#### Overview

In exploring the theoretical concepts to inform the research design, the initial focus was on the health informatics competencies required for health service managers to perform effectively in today’s health care environment, where the use of health informatics and digital tools is the norm [19-23]. Accordingly, preparatory work was undertaken to first explore competencies and what health informatics competencies already existed within the Australasian College of Health Service Management (ACHSM) Master Health Service Management Competency Framework competencies [1] and in the Australian Health Informatics Competency Framework (AHICF) [30], which apply to health service managers in Australia. It should be noted that in the 2022 revision of the AHICF, an international health informatics frameworks review was included that examined changes in the competency frameworks from the United Kingdom, Canada, Saudi Arabia, and the United States.

Second context, health service managers must be able to deliver these competencies in the context of health services management. Third competency (effective performance), health service managers need to be able to demonstrate competency, so what approach is recommended to measure the attainment of health informatics competencies for health service managers?

Digital competence and the demonstration of these competencies are context-sensitive [27,28]. Understanding the requisite knowledge and skills that are necessary to ensure the development of competency in the digital health environment is crucial, particularly in terms of rapid digital transformation. In addition, structural enabling and inhibiting factors to support a health service manager’s demonstration of digital competence must be addressed to ensure the efficacy of this competency. Underpinning this understanding is the importance as to how the development of competence can be supported at the organizational level as well as understanding the challenges health service managers face in demonstrating their competence in the digital context. This understanding must be informed by the system level and organizational factors that can support and enable the development and demonstration of competence in this dynamic, digital environment. Consequently, this preparatory work and the initial findings informed the significance of the research study and the final study design.

#### Research Focus, Research Questions, and Intended Benefits

The original focus of the research was “improving health informatics competencies for health service managers in Australia.” The research project was designed in 2019. Since the COVID-19 pandemic, significant developments in digital health have been witnessed. Following the in-depth literature review, ongoing research work, and prevailing industry activities with the Australasian Institute of Digital Health (AIDH), this highlighted that digital health had now become the umbrella term covering health informatics. Digital health is becoming increasingly relevant in the contemporary environment and is the more commonly used lexicon in the industry. The competency frameworks now being developed internationally are increasingly focusing on digital health rather than health informatics alone [31].

The Australian Digital Health Agency [18] has also identified that “digital health is increasingly recognised as an area for research, capability development and investment” [18]. Therefore, expanding the study from health informatics only, to all aspects of digital health, was more relevant to the current health care context and climate.

The research study will contribute to the development of the capability for health service managers in managing health services in the digital health era and enabling digital transformation in the health context by seeking answers to the following three research questions: (1) To what extent can the existing health service management and digital health competency frameworks guide the development of competence for health service managers in understanding and managing in the digital health space? (2) What are the competencies that are necessary for health service managers to acquire in order to effectively work with and manage in the digital health context? (3) What are the key factors that enable and inhibit health service managers to develop and demonstrate digital health competence in the workplace?

## Methods

### Phase 1

The following initial 2 steps were completed in order to confirm the significance of the study and study design and to develop a conceptual framework as a research guide to answer the 3 research questions.

#### Step 1: Competency Mapping

Cognizant of the existing and enduring efforts to support health management competency development in the digital health context, the purpose of this initial step was to comprehend existing structures and factors that can enable competency development and education in digital health management. This guided the primary investigation enumerating the extent to which postgraduate health care management education in Australian universities facilitates the development of informatics competencies. A health care management and informatics competency mapping process was undertaken using industry certification competency frameworks to identify, collect, map, and then distinguish the competencies evidenced in the postgraduate health care management programs. As the leading health informatics competency framework in Australia, the 50 health informatics competency statements from the AHICF were first mapped to the ACHSM Master Health Service Management Competency Framework competencies [1]. These were then mapped to the 21 ACHSM accredited, and the Royal Australasian College of Medical Administrators (RACMA) recognized, postgraduate health care management programs offered domestically in Australia. The ACHSM accredits, and RACMA approves the health care management degrees in Australia. These are voluntary processes that are seen as the recognized industry standard and acknowledgment for higher degrees for health service managers. The mapping process used the “Steps Used to Effectively Map Preexisting Courses to Competency Sets” approach created at the University of Washington School of Public Health’s Northwest Center for Public Health Practice, as it has been shown to have a prominent level of assurance in the accuracy of the competency mapping process for university courses [32,33].

Based on the Association of American Medical Colleges’ Curriculum Management and Information Tool (CurrMIT) [34], the Competency Mapping Matrix spreadsheet was constructed, which contained the program names, subject or course topic learning objectives, and the 50 AHICF competencies. Each of the 21 postgraduate health care management programs was then reviewed by subject title, learning objectives, and assessments for keywords, competency indicators, and themes that matched with the competency framework. The degree to which the competency was seen as being addressed was then mapped using a four-point criteria scale: (1) not addressed, (2) partially addressed, (3) mostly addressed, and (4) fully addressed. To confirm the reliability and validity of the rating instrument, piloting of the Competency Mapping Matrix spreadsheet was completed with 1 academic and 1 professional services Australian university staff member, both working within health care management postgraduate awards.

An experienced international health informatics academic completed the primary mapping, with a health service management competency specialist and management competency researcher providing oversight. Independent validation was then provided by an expert health informatics competency specialist educator and previous head of a university business school. Interrater reliability of the competency mapping process and tool was used to establish the validity of the mapping itself. Any discrepancies were resolved by a subject matter expert (an Australian professor in health service management) [35].

#### Step 2: Scoping Review

##### Overview

The second step was to conduct a scoping review of research literature to confirm the changing competency requirements for health service managers as a result of digital transformation in health care [6]. This published work explored the competencies health service managers need to acquire to effectively manage in the digital health context as well as describing the key factors that enable and inhibit health service managers in demonstrating digital health competence in the workplace. This scoping review of the literature was conducted in 2022 and built on a rapid review undertaken in 2020 [25]. The review was guided by Arksey and O’Malley’s five-step framework [36], using the following steps: (1) defining a research question, (2) identifying relevant studies, (3) selecting and confirming empirical studies, (4) data extraction, and (5) collating, summarizing, and reporting results.

##### Data Sources and Search Strategies

The following databases were searched: PubMed, Scopus, ProQuest, Web of Science, CINAHL, ACM Digital Library, Google Scholar, and ProQuest Dissertations. The following keywords were used: “digital health,” “electronic health,” “health informatics,” “competencies,” “capability,” “proficiency,” “certification,” “qualification,” “health manager,” “health executive,” and “health administrator.” A PRISMA (Preferred Reporting Items for Systematic Reviews and Meta-Analyses) approach [37] was used for eligibility screening. The review searched for publications in the English language since the year 2000 that were peer-reviewed, empirical, and from the gray literature, including government white papers and professional institution position papers.

##### Data Extraction

Data were extracted from eligible publications and included authors, year of publication, country of origin, the aim of the study, target population, methods for data collection, and key findings related to the scoping review objectives. Content analysis was performed on the extracted data to assist in developing an understanding of the content and identifying the principal themes pertinent to the search focus.

The original search conducted in 2020 generated 1212 publications, and after duplicates were removed, 941 publications were included for title screening. This led to 185 papers being included for abstract screening by 2 reviewers. The revised search conducted in 2022 generated 467 publications, and after the removal of duplicates, 403 publications were included for title screening. This led to 114 papers that were included for abstract screening, which resulted in an additional 4 papers that went through full-text review with the initial 185 papers identified. Full-text review confirmed that 81 publications were particularly relevant to digital health competencies and workforce development in the digital health context. From which, 19 papers pertinent to the focus of the scoping review then underwent qualitative content analysis [6].

### Phase 2

#### Overview

**Figure 1:**
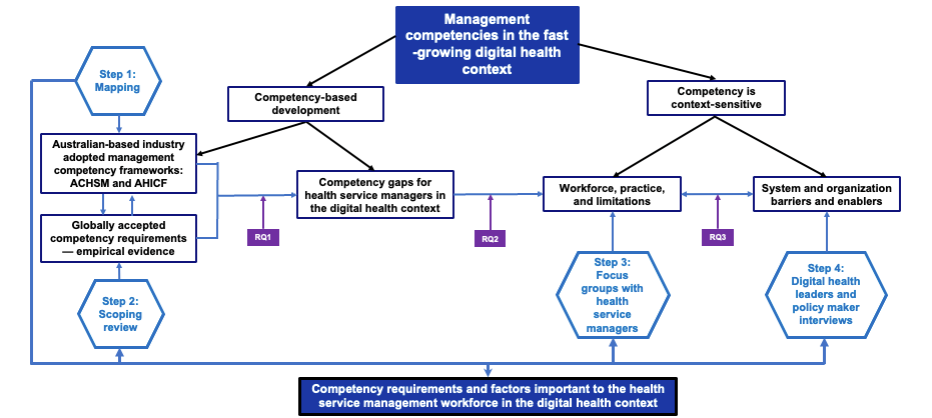
Conceptual framework—researching digital health competencies for health service managers. Blue lines show the research process. Purple boxes show the research questions (RQs). RQ1: To what extent can the existing health service management competency framework guide the development of competence for health service managers in understanding and managing in the digital health space? RQ2: What are the competencies that are necessary for health service managers to acquire in order to effectively work with and manage in the digital health context? RQ3: What are the key factors that enable and inhibit health service managers to develop and demonstrate digital health competence in the workplace? ACHSM: Australasian College of Health Service Management; AHICF: Australian Health Informatics Competency Framework.

Based on the learnings from the competency mapping, the scoping review, and the knowledge gaps identified in the literature, the conceptual framework presented in Figure 1 has been developed to guide the overall design of the study. The framework follows the research process steps (the blue lines) and addresses the 3 research questions (the purple boxes). The study has adopted a qualitative approach, guided by the empirically validated management competency identification process [27], using the following four steps: (1) health management and digital health competency mapping, (2) scoping review of literature and policy analysis, (3) focus group discussions (FGDs) with health service managers, and (4) semistructured interviews with digital health leaders. This approach is also substantiated by consistent evidence in the international literature supporting the use of these data collection methods in identifying and analyzing informatics and digital competencies in health care [38-41].

As mentioned earlier, the first 2 steps have been completed as part of the preparation process, and the overall results from these 2 steps provided the foundation for and informed steps 3 and 4 of this research study. Data collection and analysis, including triangulation of data, will be completed by December 2023. Table 1 details the target populations and the proposed number of participants for steps 3 and 4.

**Table 1:** Details of the qualitative research study focus group discussions and interviews.

Methods	Focus group discussions via videoconference	Semistructured interviews via videoconference
Focus	To explore the competencies that health service managers need to acquire to effectively work with and manage in the digital health context, along with factors that enable and inhibit the acquisition of these competencies.	To validate findings from the focus group discussions in relation to health service manager competencies, barriers, and enablers for managing in the digital health environment. Also exploring issues that influence digital health policy and practice.
Who are the participants?	Mid-level managers who are responsible for the day-to-day operations of Australian public hospitals. These positions may include department directors, unit managers, and ward managers representing level 3 and 4 management^a^ from public hospital settings.	Digital health leaders and chief digital health or clinical information officers from national digital health organizations and public health departments who have executive responsibility for digital health.
Participants, n	48 (8 per group×6 focus groups)	15

^a^Management levels are defined by the organizational reporting hierarchy, with level 1 being the chief executive officer, level 2 reporting to the chief executive officer, level 3 reporting to level 2 management, and level 4 reporting to level 3 management.

#### Sampling

A purposive sampling technique will be used to identify participants for both the focus groups and interviews, as it enables a deliberate selection of a participant due to their characteristics linked to the study variables. This sampling “involves identification and selection of individuals or groups of individuals that are proficient and well-informed with a phenomenon of interest” [42]. Furthermore, snowball sampling will be used to assist in locating additional key informants who are information-rich and possess a certain characteristic of research relevance with the target population.

#### Recruitment

##### For the FGDs

Potential participants for the FGDs will be invited and recruited from the members of AIDH membership or ACHSM. Invitations of participation will be announced by AIDH and ACHSM in their newsletters and websites. The principal investigator’s contact details will be included in the announcement. The announcement will encourage and invite potential participants to contact the principal investigator directly to express their interest to participate. Upon receipt of the expression of interest, the principal investigator will assess the eligibility and contact eligible participants directly via email to provide a formal invitation to participate in the FGD, which will contain a copy of the project information sheet and consent form. The principal investigator will also email those who expressed their interest in participating and who were not eligible and provide them with an explanation as to why they were not selected.

##### For the Semistructured Interviews

The semistructured interview participants will be invited and recruited from the members of the Digital Health Executive Network AIDH membership or ACHSM membership. Invitations of participation will be announced by AIDH and ACHSM in their newsletters and websites. The principal investigator’s contact details will be included in the announcement. The announcement will encourage and invite potential participants to contact the principal investigator directly to express their interest to participate. Upon receipt of the expression of interest, the principal investigator will then assess the eligibility and contact eligible participants directly via email to provide a formal invitation to participate in the semistructured interview, which will contain a copy of the project information sheet and consent form. If there is no response within 5 working days, 1 further email will be sent before the completion of 1 follow-up phone contact. The principal investigator will also email those who expressed their interest in participating and who are not eligible and provide them with an explanation for not being selected.

#### Ethical Considerations

This research received ethics approval on October 20, 2022, by the James Cook University Human Research Ethics Committee (approval H8877; expiry December 27, 2024). For the focus group and interview participants who respond to the AIDH or ACHSM expression of interest to participate in the general announcement and meet the health service managers’ inclusion criteria, an invitation email will be sent to potential participants by the PhD candidate with the participant information sheet and consent form attached to the email. Participants are asked to complete, sign, and return the consent form if they would like to attend the FGD or semistructured interview. The personal information collected will include participant name, position title, name of organization, and email address; however, no names will be retained, participants’ identity will not be disclosed, and all information is nonidentifiable. Each participant will be provided with a copy of their FGD or interview transcript, and they can request any specific information in the transcript to be excluded from the analysis. Results of the study will be presented collectively, and no individual information will be used. Participants will volunteer 90 minutes of their time to participate in the FGD or 45 minutes of their time to participate in the semistructured interview; there will be no payment to participate in the research study.

#### Conducting FGDs and Semistructured Interviews

In total, 6 FGDs will be conducted via videoconference (Microsoft Teams; Microsoft) by the principal investigator and 1 other member of the research team, who will play the role of observer or notetaker. The participants will be 40-50 midlevel managers who are responsible for the day-to-day operations of Australian public hospitals. These positions include department directors, unit managers, program managers, and ward managers, representing level 3 and 4 management from public hospital settings. Management levels are defined by the organizational reporting hierarchy, with level 1 being the chief executive officer, level 2 reporting to the chief executive officer, level 3 reporting to level 2 management, and level 4 reporting to level 3 management. The number of participants is consistent with the qualitative methodology being used to ensure data saturation [43-45]. The FGDs will be conducted for approximately 90 minutes each and digitally captured via the recording functionality of the Microsoft Teams videoconference call. The automatically transcribed text will then be reviewed and proofread for language and readability to provide a full transcript of the discussions.

In total, 15 semistructured interviews will be conducted by the principal investigator via videoconference (Microsoft Teams) with digital health leaders and chief digital health or clinical information officers from national digital health organizations and public health departments, who have executive responsibility for digital health, including authority over all aspects of a significant area of work and accountability for making decisions critical to the organization’s success. The number of participants is consistent with the qualitative methodology being used to ensure data saturation [43-45]. Five open-ended questions will guide the interviews. The key informant interviews will be conducted for approximately 45 minutes each and digitally captured via the recording functionality of the Microsoft Teams videoconference call.

For both types of interviews, a person-specific transcription is provided to each participant to amend or delete any of their comments, particularly for validation of accuracy or to clarify a statement, should they wish to do so.

#### Data Analysis for the FGDs and Semistructured Interviews

All transcripts will be subsequently analyzed using NVivo software (QSR International) or Leximancer software (Leximancer) or other content analysis tools. For the focus group interviews, a thematic analysis will be conducted iteratively with data from the focus groups to develop concepts, categories, and themes. Competencies will then be analyzed and categorized against existing national and international health service management and digital health competency frameworks. Enabling and inhibiting factors will also be analyzed using Braun et al [46] 6-phase reflexive thematic analysis approach to identify, organize, analyze, and advance insight into themes or categories of the factors emerging.

Importantly, reflexive thematic analysis enables the researcher to highlight meaning as contextual or situated while simultaneously undertaking analysis of the text for meaning [47,48]. Further, the legitimacy and validity of the researcher’s subjectivity are not just acknowledged, but it is also an intrinsic asset, as reflexive thematic analysis supports the active role of the researcher in the knowledge generation process [47]. Using the 6-phase reflexive thematic analysis approach as detailed in Textbox 1 augments the acquisition of text patterns and key terms to reveal patterns that were previously unknown and furnish information with meaning [49].

**The reflexive thematic analysis 6-phase approach guiding data analysis [46].**

**Familiarization**

Moving from data generation to analysis, that is, appreciating the data, by becoming immersed in and connecting with the data in different ways.

**Generating codes**

Moving to a more in-depth and systematic engagement with the data, that is, making sense of the data, by curating a list of terms, filtering out textual noise, and generating clear patterns to extract more valuable insights [49].

**Constructing themes**

Moving from codes into overarching themes that accurately and coherently represent the data, that is, creating a story about the data, through latent semantic analysis and singular value determination to uncover themes [49].

**Revising themes**

Moving to candidate themes and reviewing them to see how each theme relates to the others, that is, telling the overall story about the data.

**Defining themes**

Moving to clear definitions of each theme by elucidating the essence and scope of each theme, that is, what is meaningful about the data.

**Producing the report**

Moving to a final test of how well the themes work, both individually in relation to the data set and overall, that is, a logical story with sufficient evidence that the themes are relevant to the data.

The semistructured interview transcripts will then be analyzed using reflexive thematic analysis to triangulate the findings with the themes emerging from the FGDs. This will assist in validating the health service manager’s competencies required to manage in the digital health context, as well as generating perspectives on both role development and enhancement needs for a health service manager’s digital health competency, and aspirational and professional development requirements for health service managers to lead in the digital health space.

Both the FGDs and semistructured interviews will use the COREQ (Consolidated Criteria for Reporting Qualitative Research) checklist to “promote explicit and comprehensive reporting of qualitative studies (interviews and focus groups)” [50].

## Results

Results from phase 1 of the study, which commenced in October 2019, were published in July 2021 [35] and October 2022 [6] and informed the significance of the research study and the final study design, which are presented here.

### Competency Mapping Results

By completing the mapping of the 85 ACHSM Master Health Service Management Competency Framework competencies to the 50 health informatics competency statements from the AHICF, it was found that nearly half of this framework’s competencies were only partially or not addressed at all by the ACHSM framework. As the ACHSM framework guides accreditation of postgraduate health care management programs offered domestically in Australia and forms part of the RACMA fellowship program, this indicated that the existing formal graduate-level education may not be adequate in developing health service managers’ competency in the digital health era [35]. This published work highlighted an important gap to address—developing skills for the health management workforce relies on industry competency framework requirements that guide the curriculum and training endeavors as well as for the university programs, professional organizations, and individual health care institutions.

### Scoping Review Results

The scoping review confirmed the five key activities that influence the development of health management workforce competency and capacity, namely: (1) using competency assessment, (2) guided by competency models, (3) supporting formal professional development, (4) including short-term training activities, and (5) using work-based development. Additionally, the following seven key factors that facilitate development of the health management workforce in the digital health context were also identified and included: (1) acknowledgment and recognition of these new digital responsibilities, (2) the organization’s capacity to adopt innovation in a supportive environment to embrace digital health, (3) system-level support and political will, (4) specialized digital technology expertise, (5) an investment in the health service management workforce, (6) being underpinned by systematic integration in planning, and (7) being used for practice development [6]. How these key activities work in the health care context and how these factors should be addressed require further exploration.

## Discussion

### Anticipated Findings

#### Focus Group Discussions

The FGD participants will provide reflections and insights to explore the competencies that health service managers need to acquire to effectively work with and manage in the digital health context along with factors that enable and inhibit acquisition of these competencies. The FGDs will explore the relevance of the additional competencies identified in steps 1 and 2. Findings of the identified list of important competencies from the FGDs will then inform the finalization of the semistructured interview questions.

#### Semistructured Interviews

The focus of the semistructured interviews is to explore (1) validation of the findings from the FGDs in terms of core health service manager competencies, barriers, and enablers to manage in the digital health environment and (2) in-depth insights affecting digital health influences on digital health policy and practice.

### Limitations

It is important to note some limitations associated with a research protocol. The study is qualitative in nature to develop an in-depth understanding of Australian health service manager’s experiences and views. The methods described could be applied in other contexts; however, the results may not be generalized to other country contexts. Any recommendations provided in subsequent publications will note these limitations, and suggested findings are contextualized to individual jurisdictions and environments.

### Industry Impact

Review of the AHICF competencies was completed by the Certified Health Informatician Australasia Examination Committee in 2022, of which the principal investigator is a member, and version 2.0 of the AHICF [51] now contains 11 leadership and management competencies in domain D, up from the previous 5 management science competencies in domain 4 of version 1.0.

Further, a review of the ACHSM Master Health Service Management Competency Framework was completed by the National Steering Committee in 2022, of which the principal investigator was a member, and version 2.0 [52] now contains 6 digital management competencies in an action domain, whereas no specific digital competencies were articulated in version 1.0.

### Conclusions

This study will provide real-time, in-depth insights into the health service management workforce performance and development needs for digital health and will uniquely inform credentialing and professional development requirements for health service management. Further, this will also illuminate the postgraduate programs and continuing professional development requirements for health service management education and training. Developing strategies based on these findings will assist in supporting the health service managers to lead and manage the adoption and implementation of digital health technology and initiatives in the health care sector, including insights into the health care system’s digital workforce policy and capacity considerations. This will assist in optimizing the outcomes from adoption of digital health as a contemporary tool for health care delivery and furthering digital transformation outcomes for patients, practitioners, providers, and the public.

## Abbreviations

ACHSM: Australasian College of Health Service Management
AHICF: Australian Health Informatics Competency Framework
AIDH: Australasian Institute of Digital Health
COREQ: Consolidated Criteria for Reporting Qualitative Research
FGD: focus group discussion
HIPAA: Health Insurance Portability and Accountability Act
PRISMA: Preferred Reporting Items for Systematic Reviews and Meta-Analyses
RACMA: Royal Australasian College of Medical Administrators

## Data Availability

The data sets generated during and analyzed during this study are not publicly available due to ongoing data collection from participants but may be made available from the corresponding author on reasonable request.
